# Fine Tuning of a Type 1 Interferon Antagonist

**DOI:** 10.1371/journal.pone.0130797

**Published:** 2015-07-09

**Authors:** Victoria Urin, Doron Levin, Nanaocha Sharma, Daniel Harari, Gideon Schreiber

**Affiliations:** Department of Biological Chemistry, Weizmann Institute of Science, Rehovot, 76100, Israel; Schulze Center for Novel Therapeutics, Mayo Clinic, UNITED STATES

## Abstract

Type I interferons are multi-potent cytokines that serve as first line of defense against viruses and other pathogens, posses immunomudolatory functions and elicit a growth inhibitory response. In recent years it has been shown that interferons are also detrimental, for example in lupus, AIDS, tuberculosis and cognitive decline, highlighted the need to develop interferon antagonists. We have previously developed the antagonist IFN-1ant, with much reduced binding to the IFNAR1 receptor and enhanced binding to IFNAR2. Here, we further tune the IFN-1ant by producing three additional antagonists based on IFN-1ant but with altered activity profiles. We show that in all three cases the antiproliferative activity of interferons is blocked and the induction of gene transcription of immunomudolatory and antiproliferative associated genes are substantially decreased. Conversely, each of the new antagonists elicits a different degree of antiviral response, STAT phosphorylation and related gene induction. Two of the new antagonists promote decreased activity in relation to the original IFN-1ant, while one of them promotes increased activity. As we do not know the exact causes of the detrimental effects of IFNs, the four antagonists that were produced and analyzed provide the opportunity to investigate the extent of antagonistic and agonistic activity optimal for a given condition.

## Introduction

Type 1 Interferons (IFNs), discovered more than half a century ago are part of the helical cytokines superfamily [1]. They are secreted proteins that are essential for antiviral (AV) immunity, antiproliferation (AP) and immunomodulatory activities in vertebrates [2, 3], acting in practically every nucleated cell. Due to their wide range of activities type 1 IFNs are used as a treatment of various human diseases, such as multiple sclerosis, hepatitis C and cancer [4, 5]. In humans the family consists of 16 members: 12 IFN-α subtypes, IFNβ, IFNω, IFNκ and IFNε. All type 1 IFNs bind the two common cell surface receptor components, IFNAR1 and IFNAR2 [6–8] followed by activation of the intracellular JAK (janus kinase) /STAT (signal transducers and activators of transcription) pathway. Upon complex formation, the tyrosine kinases Tyk2 and Jak1, which are constitutively associated with the IFNAR1 and IFNAR2 subunits, respectively, activate each other by phosphorylation, and then phosphorylate key tyrosine residues located in the IFNAR1 and IFNAR2 intracellular regions [9, 10]. Subsequently, STAT1 and STAT2 proteins are therein recruited and tyrosine-phosphorylated in order to translocate into the nucleus and form, together with IRF9, the ISGF3 transcriptional complex driving the expression of IFN-stimulated genes [9]. Type 1 IFNs activate a variety of genes, some of which require low concentration (pM) of IFN and short term induction. Those genes are referred as “robust” and they mediate the AV activity. Other genes demanding high concentrations of IFN (nM) for longer induction periods mediate the AP abilities and are referred to as “tunable” genes [11].

Despite their genetical and structural similarity, the various type 1 IFNs vary significantly in their abilities to induce gene expression and consequently in their AV and AP potencies, clinical responses and in stimulation of immunoregulatory responses [12]. These differences are largely attributed to the different binding affinity towards the receptors. IFNα1 is the weakest binder, the other IFNα’s are intermediate binders and IFNβhas the highest binding affinity towards both receptors [13]. This was confirmed by generating tighter binding IFN mutants, such as the YNS, (N57Y, E58N and Q61S), [14], which combined with the carboxyl-terminal eight amino acids (α8tail) found in IFNα8 binds 60-fold tighter to IFNAR1 and 15-fold tighter to IFNAR2 than IFNα2, even surpassing the binding affinity of IFNβ. The antiproliferative potency of YNS-α enhanced accordingly [11]. On the other side of the spectrum, a type 1 IFN antagonist (IFN-1ant) was generated by mutating the Arg 120 to Glu and adding the α8-tail [15], thereby reducing its affinity to IFNAR1 to below detection level while increasing the affinity to IFNAR2. At high (100 nM) concentration, IFN-1ant manages to elicit an antiviral response equals to that elicited by ~1 pM of WT IFNα2, a concentration sufficient to partially activate the antiviral action but not the antiproliferative response [16]. At these high concentrations IFN-1ant is also an inhibitor, inhibiting the production of tunable genes and the immunomodulatory and antiproliferative responses of other type I IFNs.

Here, we engineered three new antagonists on the basis of IFN-1ant with varied activity profiles. The locations of the new mutations were chosen from a previous alanine scan, which determined positions 65 and 85 on IFNα2 as important for binding to IFNAR1 [17]. We show that N65 to A or Y85 to M reduced the antiviral activity to bellow that of IFN-1ant, while adding the Y85A mutation resulted in an increased antiviral potency compared to IFN-1ant. Still, none of these variants promotes an antiproliferative response and all the antagonists exhibited competitive antagonistic properties in combination with IFNα2. Finally, Robust and tunable genes were characterized in a number of cell lines. Examination of gene expression further verified that different transcriptional responses mediate the antagonists activities. With the newly found awareness of the detrimental effects of having too much type I IFN in the system, the fine tuning of the IFN antagonists may be of great value to bring interferon under control in cases such as lupus, tuberculosis, AIDS and cognitive decline [18–21].

## Materials and Methods

### Cell lines

WISH is a human amniotic epithelial cell line. The T47D breast cancer cell line and the OVCAR3 ovarian cancer cell line are part of the NCI60 panel of human tumor cells. WISH cells were grown in Minimal Essential Media with 10% Fetal bovine serum (FBS), 1% L-glutamine and 1% Penicillin-Streptomycin (Pen/Strep), OVCAR3 and T47D cells were grown in Roswell Park Memorial Institute medium (RPMI-1640) with 10% FBS, 1% L-glutamine and 1% Pen/Strep.

### Protein expression and purification

Recombinant IFNs and IFNARs were expressed using the *Escherichia Coli* strain BL21 (DE3) and Rosseta and purified as described previously [14].

### In vitro binding assay

The mutant and wild type IFNs binding affinity towards IFNAR1-EC and IFNAR2-EC were determined by SPR, using the ProteOn XPR36, as detailed previously [11]. The dissociation constant K_D_ was obtained from the equilibrium response as previously described [17].

### In situ binding assay

Wild-type IFNα2 was labeled with ^125^I by PheonixPepdide (USA). For the competition assay WISH cells were grown on 24-well plates, washed once with PBS + 0.1% sodium azide, and then incubated for 10 min with the same solution. Next, cells were incubated for 1 hr at room temperature with the labeled wild type IFNα2 (200,000 cpm/well) in the presence of an unlabeled IFNα2 of interest at ten different concentrations: from 200 nM for all the antagonists and 10 nM for the IFNα2-α8tail at 4-fold dilution steps in culture medium + 0.1% sodium azide. Cells were then washed five times in PBS on ice to get rid of unbound interferons, trypsinated, and transferred into test tubes for measuring of bound, ^125^I-labeled IFNα2, using a γ-counter (Packard). IC_50_ values were calculated using Kaleidagraph Synergy Software.

### Antiviral and antiproliferative assays

1.2x10^4^ WISH or OVCAR3 cells were grown on flat-bottomed 96-well plates for the antiviral assay, and 7x10^3^ for the antiproliferative assay over night. Cells were treated with 20 serial dilutions of IFNs or with two concentrations (1000nM and 100nM) for AV and AP assays respectively. AV activity against VSV and EMCV were assessed by determining the extent of viruses’ cytophatic effect inhibition. Four hours after the addition of IFNs, VSV or EMCV were added for 17 or 23-hours incubation respectively. AP was monitored 72 hours after the addition of IFNs. In both assays the cell viability was determined by crystal violet staining. The EC_50_ values and cell sensitivity to the indicated IFNs were determined from the IFN dose-response curve, as previously described [17].

XTT cell viability assay (Biological Industries) is based on mitochondrial enzymatic activity, which reduce XTT to orange colored product. The intensity of the orange color is proportional to the number of living cells in the sample.

### pSTAT1 and pSTAT2 analysis

STATs phosphorylation was measured by Western blot as described earlier [11] using the following antibodies: polyclonal anti-pSTAT1 (Tyr701) (Santa Cruz Biotechnology Inc.), polyclonal anti-pSTAT2 (Tyr689) (Millipore), polyclonal anti-STAT1 (Santa Cruz Biotechnology Inc.), polyclonal anti-STAT2 (Delta Biolabs), and monoclonal anti–α-tubulin (Sigma). Quantitative analysis of band intensities on Western blots was performed with Image Studio Lite software.

### Quantitative PCR analysis

The extent of expression of human IFN–stimulated genes was measured as described previously [11]. High-throughput qPCR was performed with BioMark 96 × 96 Dynamic Array (Fluidigm Corporation) according to manufacturer’s protocol. cDNAs (50ng/μl) were pre-amplified with all the primers and analyzed with the BioMark real-time PCR instrument. Initial data analysis was performed with the fluidigm real-time PCR analysis software. Hierarchical clustering was performed in Partek with Pearson dissimilarity and complete linkage.

### In vivo experiment

Antagonist IFNs were tested for bioactivity directly in wild type C57BL/6 mice or in the “HyBNAR mice” a transgenic strain harboring humanized type I IFN receptors [22]. Stocks of C57BL/6 mice were purchased from Harlan Laboratories, Israel. All the mice were maintained on site in the Weizmann Institute of Science animal facilities. All mouse experiments were performed strictly according to the Weizmann Institute of Science ethics committee guidelines and permissions (IACUC permit number 02160412–3). The HyBNAR mice were each injected intraperitoneally with 100ul PBS solution containing either 1ug human IFNβ, 50ug of one of the antagonists, or a combination of the both. Control HyBNAR mice were mock-injected with PBS only. This study was repeated with C57BL/6 mice, using mouse IFNβ (1 μg). Three hours post-injection mice were sacrificed by CO2 asphyxiation, livers were removed and total RNA was prepared using Tri-Reagent (MRC-Inc), followed by cDNA synthesis (High Capacity cDNA Reverse Transcription kit; Applied Biosystems, Life Technology) and subsequent quantitative PCR (qPCR) analyses of the indicated mouse genes using a 384-well qPCR device (VIIA7 Real-Time PCR System- Life Technologies). The mouse genes MX1, OAS2, IFIT1, CXCL10 and CXCL11 were amplified and relative gene expression was determined using the reference probe HPRT1 as described [23].

## Results

### Generating a family of type 1 IFN antagonists

**Fig 1:**
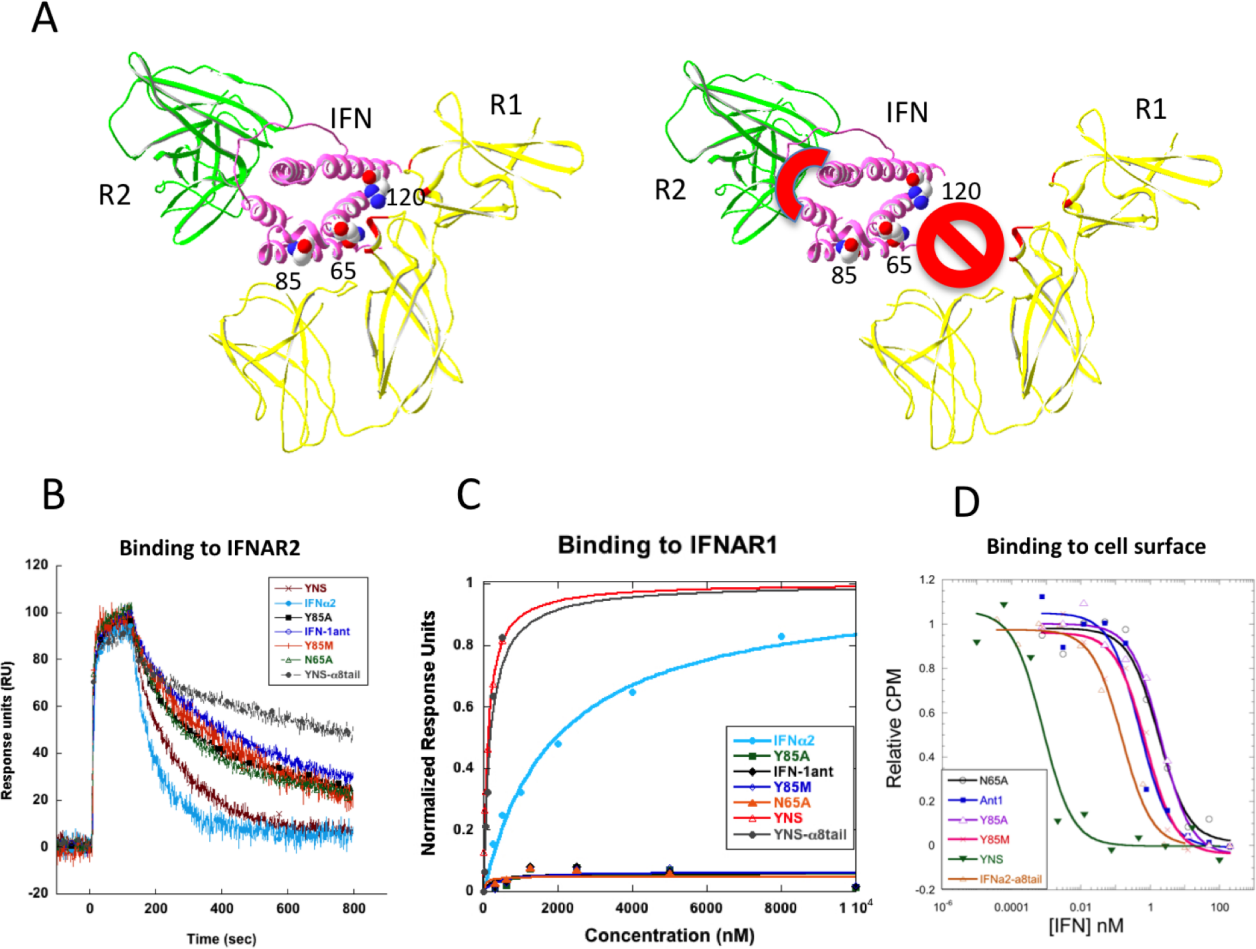
Binding of IFNα2 and its mutants to the IFNAR1 and IFNAR2 receptor subunits. (A) Ribbon representation (based on Protein Data Bank #3SE3) [29] of the ternary complex of IFNAR1 (R1), IFNAR2 (R2) and IFN, including the locations of the point mutations of the antagonists. On the right is a representation of the antagonist, which binds tighter to R2, but does not bind R1. (B) SPR sensograms of different IFNs (analyte) binding IFNAR2 (surface bound ligand) measured on a ProteOnXPR36. (C) IFNAR1-binding equilibrium analysis of the antagonists and IFNα2. The measurements were done using the ProteOn XPR36 Protein Interaction Array System (BioRad) with IFNAR1 and IFNAR2 immobilized on the sensor chip. For equilibrium binding analysis towards IFNAR1, six different concentrations of the interferon proteins were administrated, and the data were fitted using the mass action equation. (D) *In situ* measurements of the interferon antagonists towards the IFNAR receptor subunits. The 50% competition values for the four antagonists are ~1 nM, in line with their binding affinities to IFNAR2. 1000-fold lower than the EC_50_ value determined for YNS. All extracted data from panels C-E are presented in Table 1.

IFN-1ant, which harbors the IFNα2—R120E mutation and 8-C-terminal residues of IFNα8 acts as a partial antagonist to natural interferons such as IFNα2 or IFNβ [16]. IFN-1ant does not promote antiproliferative activity (independent on its concentration) and promotes a partial antiviral activity when used at high concentrations [16, 21]. Here, we produced a series of antagonists based on IFN-1ant, but including additional detrimental mutations at the IFNAR1 binding site and analyzed their activity. The three new IFN antagonists harbor the mutations N65A, Y85A and Y85M (Fig 1A). The single mutations N65A and Y85A were previously shown to reduce the *K*_D_ towards IFNAR1 by three fold [17]. The Y85M mutation was chosen as methionine is not found at this position in any type I interferon (as determined by Consurf [24]). These mutations were expected to act additively to R120E, as they are located distal from it. The new antagonist proteins were produced and purified and their binding affinities to IFNAR1 and IFNAR2 measured by Surface Plasmon Resonance (SPR), using the ProteOn XPR36 system. Comparisons of binding affinities of the various IFNα2 mutants are shown in Table 1 and Fig 1. The binding affinities of all the antagonists towards IFNAR2 are similar to that determined for YNS-α8tail (which binds ~15 fold tighter than IFNα2, see Fig 1B, Table 1 and [11]). Conversely, binding of all of the different antagonists towards the IFNAR1 chain was below the detection limit of the instrument, even when using 12 μM of the analyte (Fig 1C). *In situ* measurements of the various interferon antagonists towards the IFNAR receptors were performed by a binding competition assay on WISH cells with ^125^I-labeled IFNα2 wild-type mixed with cold interferon (either one of the antagonists, YNS, or IFNα2-α8tail). The 50% competition values for the four antagonists were 0.6–1.5 nM (Fig 1D and Table 1), in line with their binding affinities to IFNAR2. The IFNα2-α8tail had an EC_50_ value of 0.15 nM and YNS-0.001 nM. The results show that at the low surface density on WISH cells (a few hundreds per cell) ligand-binding stabilization by IFNAR1 is high for the tight binding YNS mutant [14, 25].

**Table 1:** Binding constants and antiviral response of wild-type IFNα2 and its mutants.

	IFNAR2-EC		IFNAR1-EC				
Mutation	*k*_a_ (10^6^M^-1^s^-1^)	*k*_d_ (10^-3^s^1^)	*K*_D_ (10^-9^M^1^)	*K*_D_ (10^-9^M^1^)	EC_50_ (nM)	WISH AV (Amp)	OVCAR3 AV (Amp)
IFNα2	2.6	6.7	2.6	2000	20		
YNS	2	5.5	2.8	45	0.001	0.979 (0.15)	1.0 (0.18)
YNS-α8tail	9.6	1.1	0.12	40			
IFN-1ant	5.2	1.7	0.33	ND	0.5	0.478 (0.07)	0.949 (0.09)
IFN-1ant_Y85A_	13	2.1	0.17	ND	2	0.817 (0.08)	1.0 (0.16)
IFN-1ant_N65A_	14	2.5	0.18	ND	1.7	0.297 (0.09)	0.775 (0.08)
IFN-1ant_Y85M_	18	2	0.11	ND	0.8	0.276 (0.11)	0.76 (0.1)

**Fig 2:**
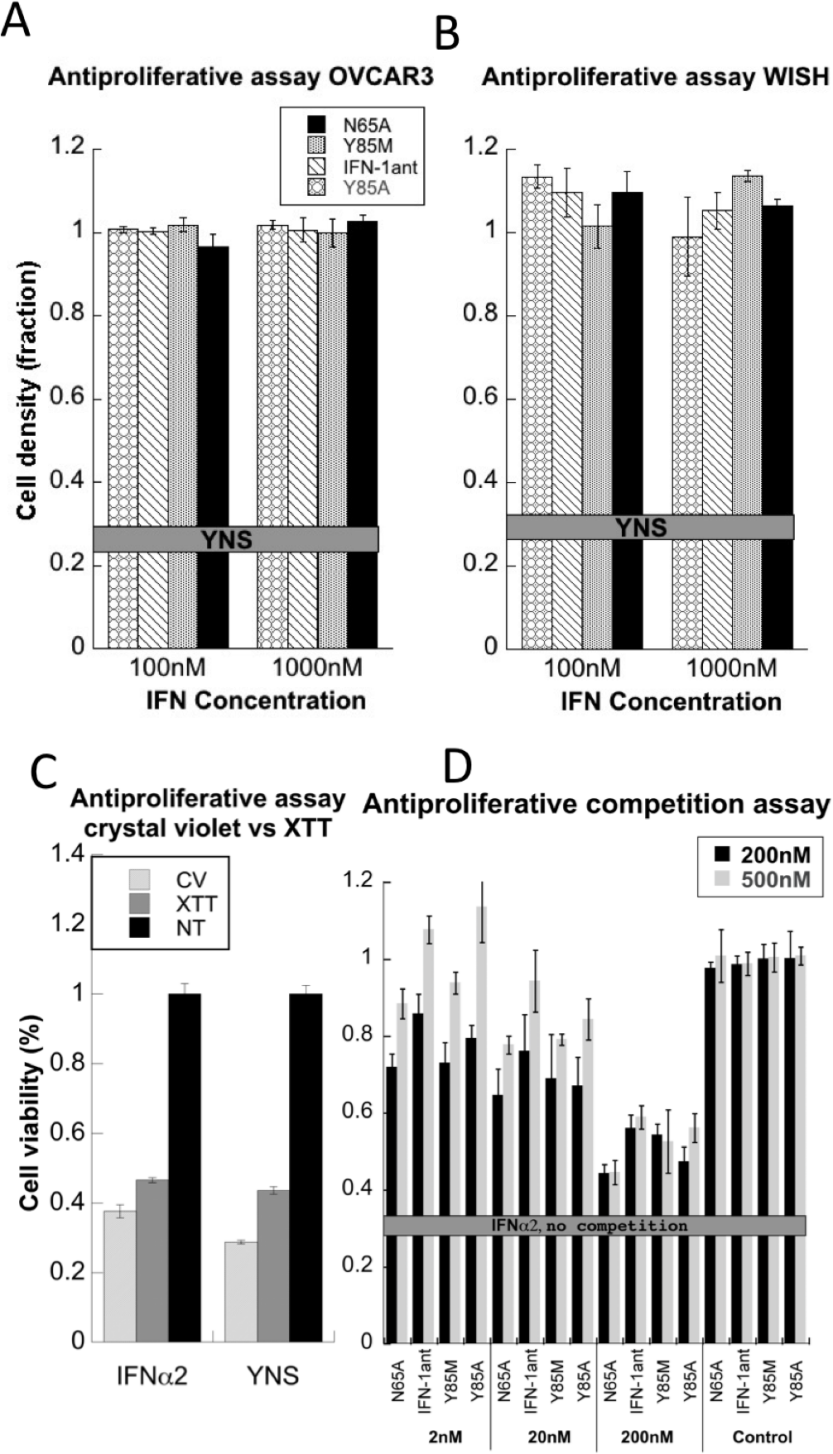
Antiproliferative activities of the type 1 IFN antagonists. Cell survival was monitored 72 hours post IFN treatment by crystal violet staining. The fraction cell density is relative to untreated cells, with the line in panels A and B indicating cell survival upon 1nM YNS treatment. (A) OVCAR3 cells, (B) WISH cells. (C) Comparing cell viability as determined by crystal violet versus the colorimetric XTT assay, using 1nM YNS and 10nM IFNα2. (D) Reduction in cell count upon treatment of WISH cells with 2, 20 or 200 nM IFNα2 together with 200 or 500 nM antagonists for 72 hours. The grey line indicates cell survival upon 10 nM IFNα2 treatment. The antiproliferative activity of each IFN alone is presented at the right side of the chart (Control). The error bars are SE from 3 independent experiments.

*k*_a_, *k*_d_, and *K*_D_ values were determined using the XPR36 from measurements of six different protein concentrations as shown in Fig 1, with the extracellular domain of IFNAR2 being the ligand. The fraction amplitude (Amp) of antiviral response (see Fig 2) and the errors (in parenthesis) were calculated from the IFN AV dose-response curves. EC_50_ values are calculated from *In situ* measurements of ^125^I labelled IFNs binding to the cell surface. Standard Error for *k*_a_ values is 25%, for *k*_d_ is 15% and for *K*_D_ is 30%.

### Blocking antiproliferative activity

The antiproliferative activity of the different interferon variants were determined using two antagonist concentrations, 100 and 1000 nM on both OVCAR3 (Fig 2A) and WISH (Fig 2B) cell lines. The AP activity of 1 nM YNS is taken as maximum activity and shown as bold line. None of the antagonists had detectable antiproliferative activity, even at the highest concentration. To verify that cell density determined by crystal violet relates to the number of living cells we compared relative crystal violet measurements upon IFN treatments with those determined from a cell cytotoxicity assay using tetrazolium based colorimetric results (XTT), and obtained similar results for both (Fig 2C). Next, the potential of the different antagonists to compete against the antiproliferative activity induced by 2, 20 or 200 nM IFNα2 was determined. Two antagonist concentrations, 200 and 500 nM were used (Fig 2D). In all these experiments the cells were treated with the various IFNs for 72 hours and then stained with crystal violet to evaluate cell survival. The addition of either one of the four antagonists resulted in an increased cell survival, with the 500 nM antagonist concentration resulting in increased competition (higher cell survival) than 200 nM. The blocking of the IFNα2 AP activity spanned from complete blocking, when using 2 and 20 nM IFNα2 to partial reduction in AP when using 200 nM IFNα2. These results prove that all four antagonists bind tightly to the IFNAR2 chain, blocking IFNα2 from forming sufficient numbers of active ternary complexes.

### Antiviral activity

**Fig 3:**
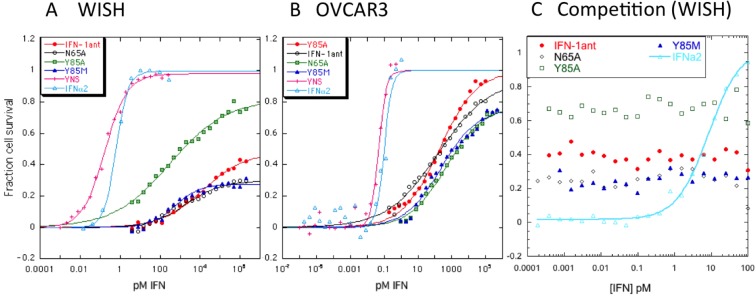
Antiviral activity of IFN antagonists in WISH and OVCAR3 cells. (A) WISH cells were treated with the antagonists, YNS, IFNα2 or left untreated for four hours. VSV virus was added for 18 hours, which after cell survival was monitored by crystal violet staining. (B) OVCAR3 cells were treated with the antagonists, YNS, IFNα2 or left untreated for four hours. EMCV virus was added for 23 hours, which after cell survival was monitored by crystal violet staining. The experiments were performed 3 times and the fitting results (for the amplitude) are reported in Table 1. (C) Reduction in antiviral activity upon combined treatment of WISH cells with 200 nM antagonists combined with 0.0001–100 pM IFNα2.

Antiviral activity of the various antagonists in WISH cells infected with VSV and in OVCAR3 cells infected with EMCV was determined. WISH and OVCAR3 cells were treated for four hours with 20 serial dilutions of the antagonists (the highest concentrations were 2 μM and 100–500 nM respectively) in comparison to YNS (highest concentration 1 nM and 10 pM respectively), which after cells were infected with VSV or EMCV and stained for survival after 17 and 23 hours respectively. On WISH cells the EC_50_ for IFN-1ant_Y85A_ was 300 pM and for IFN-1ant, IFN-1ant_Y85M_ and IFN-1ant_N65A_ ~2 nM. In comparison, the EC_50_ for YNS was 0.2 pM (Fig 3A). The more significant difference between the antagonists was the percentage of cells they managed to rescue, which varied from 80% for IFN-1ant_Y85A_ to 50% for IFN-1ant and 30% for IFN-1ant_N65A_ and IFN-1ant_Y85M_.

In general, the antiviral activity of all the antagonists was significantly stronger on OVCAR3 cells challenged with EMCV. The EC_50_ values for IFN-1ant, IFN-1ant_Y85A_ and IFN-1ant_Y85M_, and IFN-1ant_N65A_ were 170, 190, 240 and 500 pM respectively, in comparison with 0.015 pM for YNS. Again, like for WISH cells, the percent cell survival was the highest for IFN-1ant_Y85A_ (100%), 95% for IFN-1ant and 75% for IFN-1ant_Y85M_ and IFN-1ant_N65A_ (Fig 3B and Table 1).

To evaluate the potency of the different antagonists to compete the induced antiviral activity of IFNα2 we treated WISH cells with a mixture of 200 nM of each of the antagonists in the presence of 0.001–200 pM of IFNα2 in comparison to IFNα2 alone (Fig 3C). The four antagonists fully inhibited the antiviral activity of IFNα2, with the residual antiviral activity being a result of the potency of the different antagonists to induce an antiviral state (as observed in Fig 3A).

### Activation of STAT1 and STAT2 by interferon antagonists

**Fig 4:**
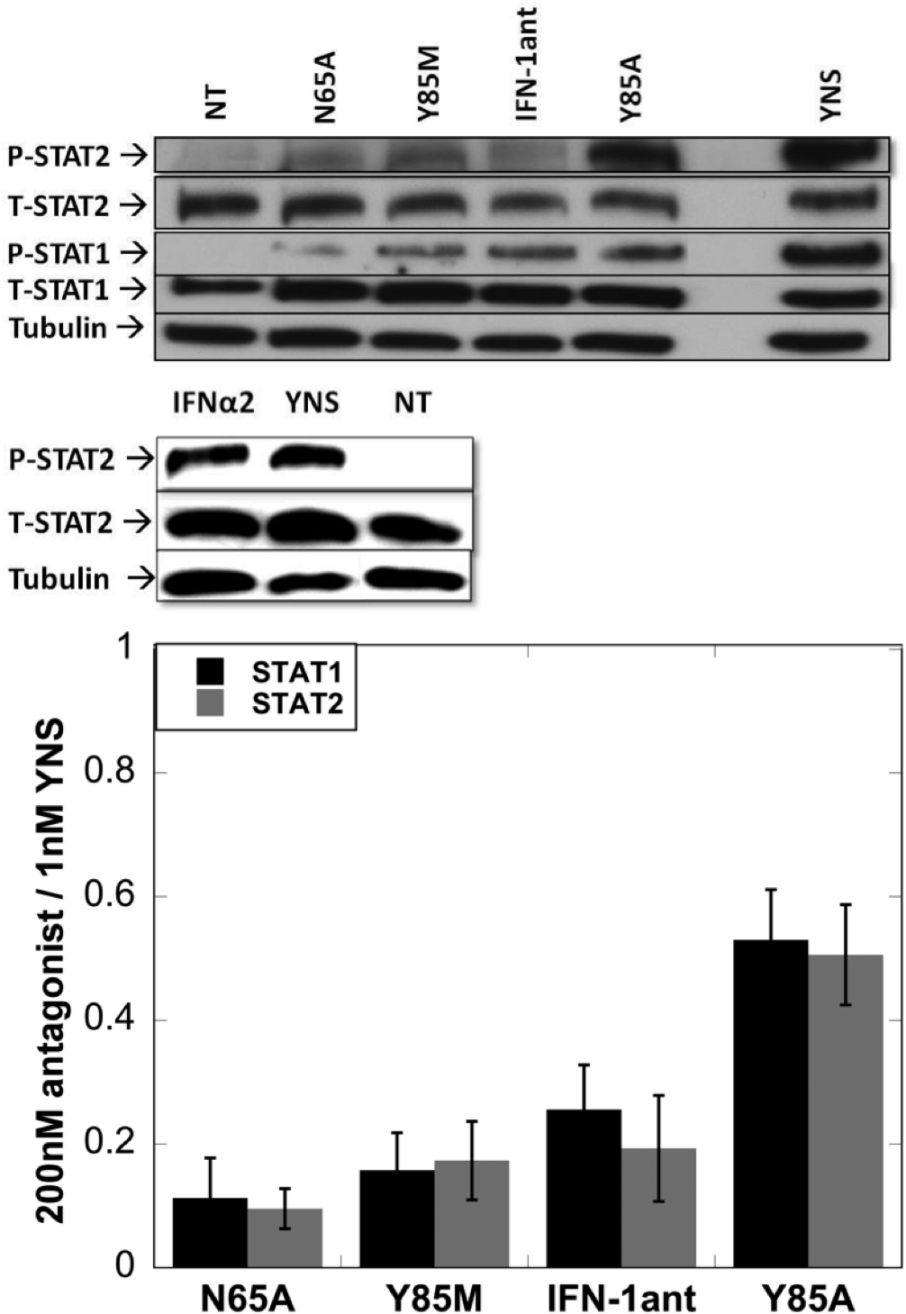
STAT1 and STAT2 activation by the IFN antagonists. WISH cells were treated with either 200 nM antagonist or 1 nM YNS for 30 minutes and analyzed for levels of STAT1 and STAT2 phosphorylation by western blot assay using specific antibodies in comparison to total protein levels. Total and pSTAT2 levels upon 1 nM of YNS or IFNα2 are shown in the middle panel. pSTAT1 levels for these two proteins were previously shown [30]. The blot was quantified using the Image Studio Lite software. The results were normalized using 1nM YNS as 100% phosphorylation and the non-treated as 0% (lower panel). The error bars are SE from three independent experiments.

Phosphorylation of STAT1 and STAT2 is key to signaling by type 1 IFNs. Levels of phosphorylation were followed after 30 min treatment with IFN (200 nM antagonists or 1 nM YNS) in WISH cells. The levels of pSTAT2 were the same for IFN-1ant_N65A_, IFN-1ant_Y85M_ and IFN-1ant and were much lower than those induced by the IFN-1ant_Y85A_ variant (which was close to that observed by treating with YNS). pSTAT1 levels following antagonist treatments were significantly lower than those obtained by YNS, with the lowest level being observed for IFN-1ant_N65A_ and the highest level for IFN-1ant_Y85A_ (Fig 4).

### Gene induction following interferon treatment

**Fig 5:**
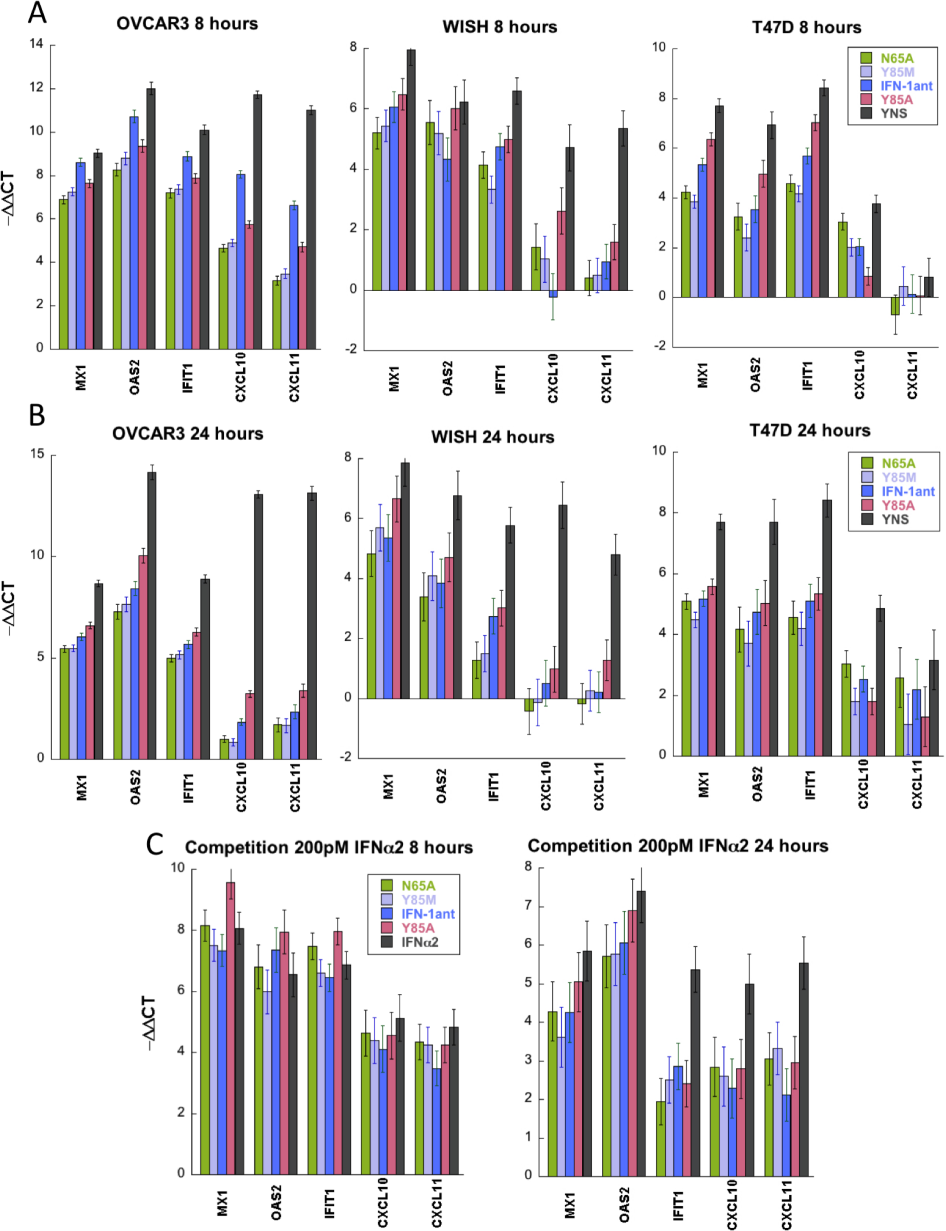
Gene expression in response to antagonist treatment. Gene expression in OVCAR3, WISH and T47D cells in response to antagonists, YNS and IFNα2. (A) Cells were treated for 8 hours with 200 nM antagonists or 1nM YNS, and analyzed by qPCR. The data presented are the relative expression levels compared to those of untreated cells, normalized against HPRT1. (B) As described in (A), but cells were treated for 24 hours. (C) Cells were treated with a combination of 200 nM antagonist and 200 pM IFMα2. Gene induction was analyzed as described in (A). Error bars represent standard deviation of the data.

Interferon induced genes can be divided into robust and tunable genes, with the robust genes being expressed already after 4 hours of low interferon treatment, while tunable genes require longer times of high interferon treatment to reach their maximum expression levels [11, 16]. Here, WISH, T47D and OVCAR3 cells were treated with 200 nM of the various antagonists or 1 nM of YNS, for 8 or 24 hours. Gene expression was evaluated by qPCR for the robust genes—MX1, OAS1, IFIT1 and tunable genes—CXCL10 and CXCL11. Overall, the antagonists promoted lower levels of gene expression than YNS. For the robust genes, inductions of 100-1000-fold were measured for the four different antagonists in three different cell lines. IFN-1ant_N65A_ and IFN-1ant_Y85M_ showed the lowest level of induction in most measurements (Fig 5A and 5B). Conversely, the antagonists promoted only very low levels of gene induction of the tunable genes after 8 or 24 hours (Fig 5A and 5B). The only antagonist promoting significant (albeit still relatively low) gene induction of the tunable genes was IFN-1ant_Y85A_. As expected, in T47D cells gene induction of the tunable genes was low even when using YNS.

Next, we measured the ability of the antagonists to suppress gene induction by IFNα2. WISH cells were treated with a combination of 200 nM antagonist plus 200 pM IFNα2. The antagonists did not suppress gene induction after 8 hours treatment, however, after 24 hours a strong antagonistic effect was observed, particularly for the tunable genes and IFIT1 (Fig 5C). The level of competition was about equal for the different antagonists. One should note that tunable genes are activated later than robust genes, thus their suppression is partially explained by their late onset.

**Fig 6:**
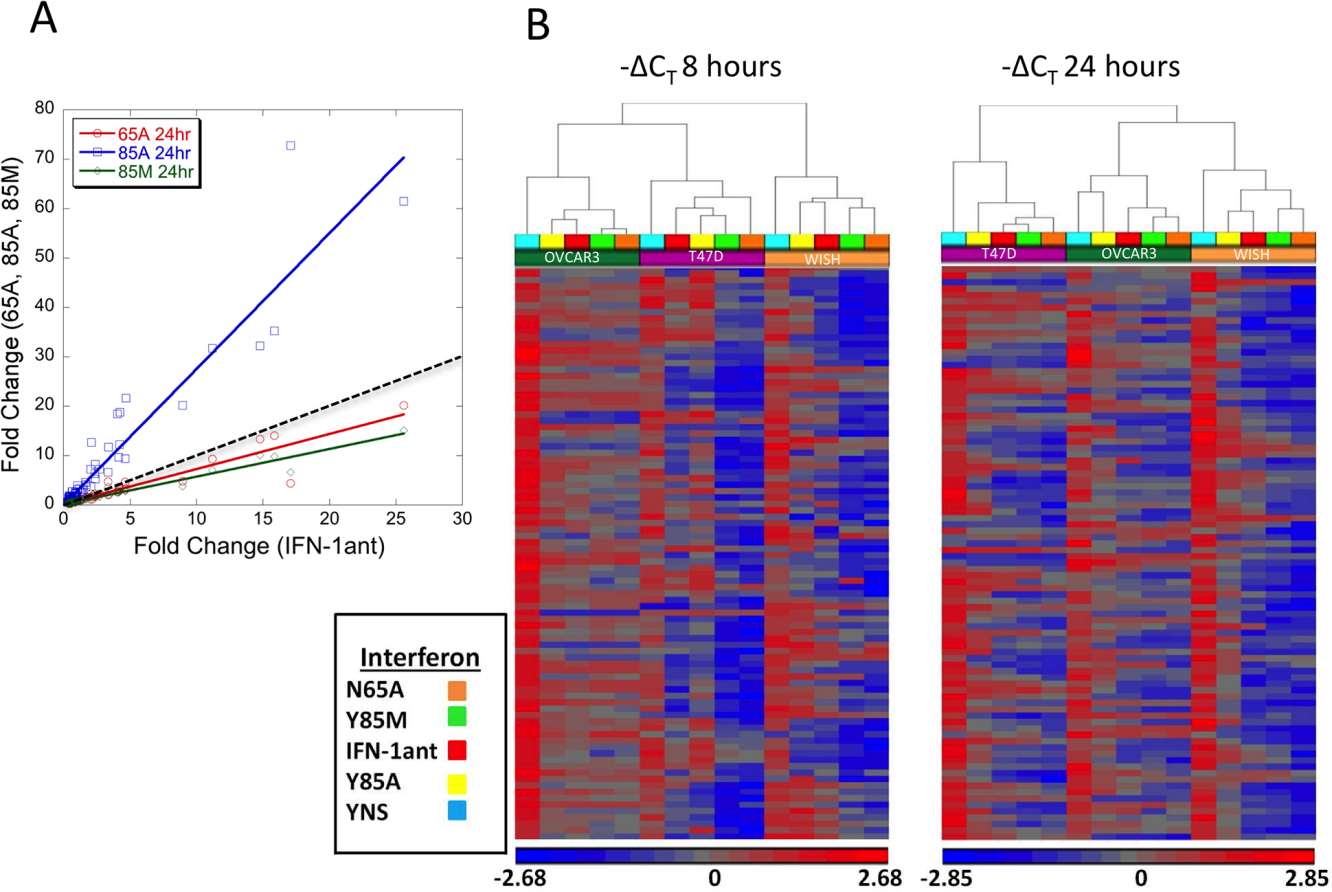
Gene induction signature upon interferon treatments. Obtaining a gene induction signature of the various IFN variants for ~100 interferon induced genes using the fluidigm system. (A) Comparison of the fold change in gene expression of WISH cells treated with 200 nM IFN-1ant versus the other antagonists. The doted line represents a slope of one. (B) Clustering analysis of -ΔC_T_ values in the indicated cell lines of gene expression data with the indicated IFNs treatments for 8 hours (left) and 24 hours (right).

To obtain a more complete picture of gene induction following the various treatments in these three cells lines we employed high-throughput qPCR on a 96 x 96 dynamic array (fluidigm). Plotting gene induction levels of IFN-1ant versus the three other antagonists gives a slope of 2.7 for IFN-1ant_Y85A_ and 0.7 and 0.6 for IFN-1ant_N65A_ and IFN-1ant_Y85M_ respectively, with no significant outlier from the linear relation (Fig 6A). The relation between gene induction levels of the different antagonists shows that at 200 nM the difference between the antagonists is quantitative and not qualitative, with IFN-1ant_Y85A_ promoting higher levels of gene induction, while IFN-1ant_N65A_ and IFN-1ant_Y85M_ promoting lower levels of gene induction than IFN-1ant. To further analyze the data, hierarchical clustering analysis was applied on the ΔC_T_ values (normalized to HPRT1) for all the different cell lines and treatments (Fig 6B). The data clearly cluster according to the cell-line at both time points. Between the antagonists, the highest gene induction is observed when treating the cells with 200 nM of IFN-1ant_Y85A_, and the lowest levels are for IFN-1ant_Y85M_ and IFN-1ant_N65A_ (both for 8 and 24 hours of treatment).

**Fig 7:**
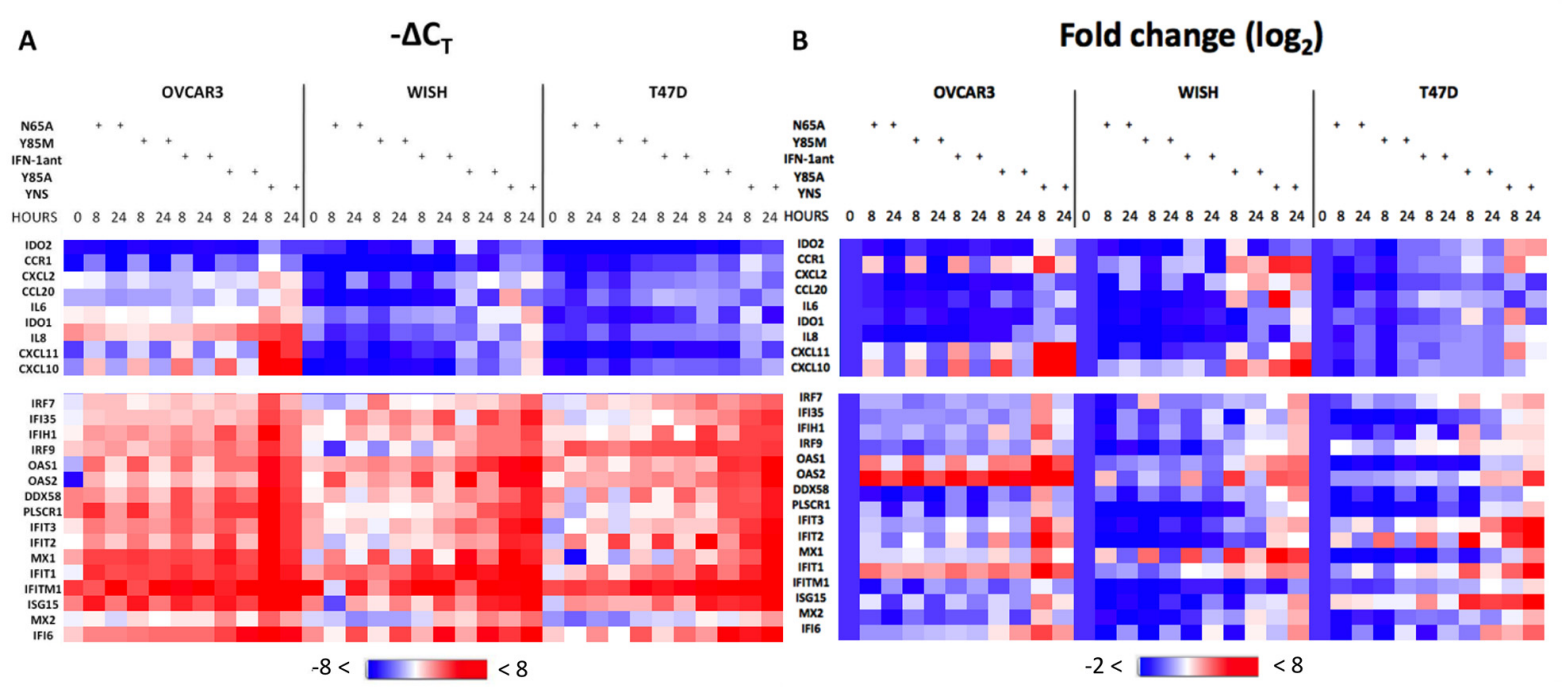
–ΔC_T_ and ΔΔC_T_ of robust and tunable genes. Comparing –ΔC_T_ (A) to ΔΔC_T_ (B) values of IFN induced tunable (upper) and robust (lower) genes. The cells were treated with 200 nM antagonists, 1nM YNS or not treated (0). The data were analyzed with the NetWalker analysis tool [31].

To obtain a more detailed view of gene induction of robust and tunable genes, we selected 16 robust and 10 tunable genes and used a heat map to compare the mRNA levels (-∆C_T_) and level of gene induction (∆∆C_T_ relative to non-treated). The ten upper genes in both the -ΔC_T_ and ∆∆C_T_ maps are of tunable genes. These include chemokines (such as CXCL10 and 11), which were shown to be involved with immunemodulatory and antiprliferative activities [26, 27] The sixteen lower genes are robust (Fig 7A and 7B). Clearly, the levels of gene expression of the robust genes are higher than of the tunable genes, as are their levels of induction in all three-cell lines. Moreover, the antagonists poorly induced the tunable genes. Consistent with previous results the level of induction of the genes in OVCAR3 cells are higher since they are very sensitive to type 1 IFNs treatments, whereas T47D cells, which are partially resistant to IFNs show significantly lowers levels of gene induction.

### Antagonist activity in mice

**Fig 8:**
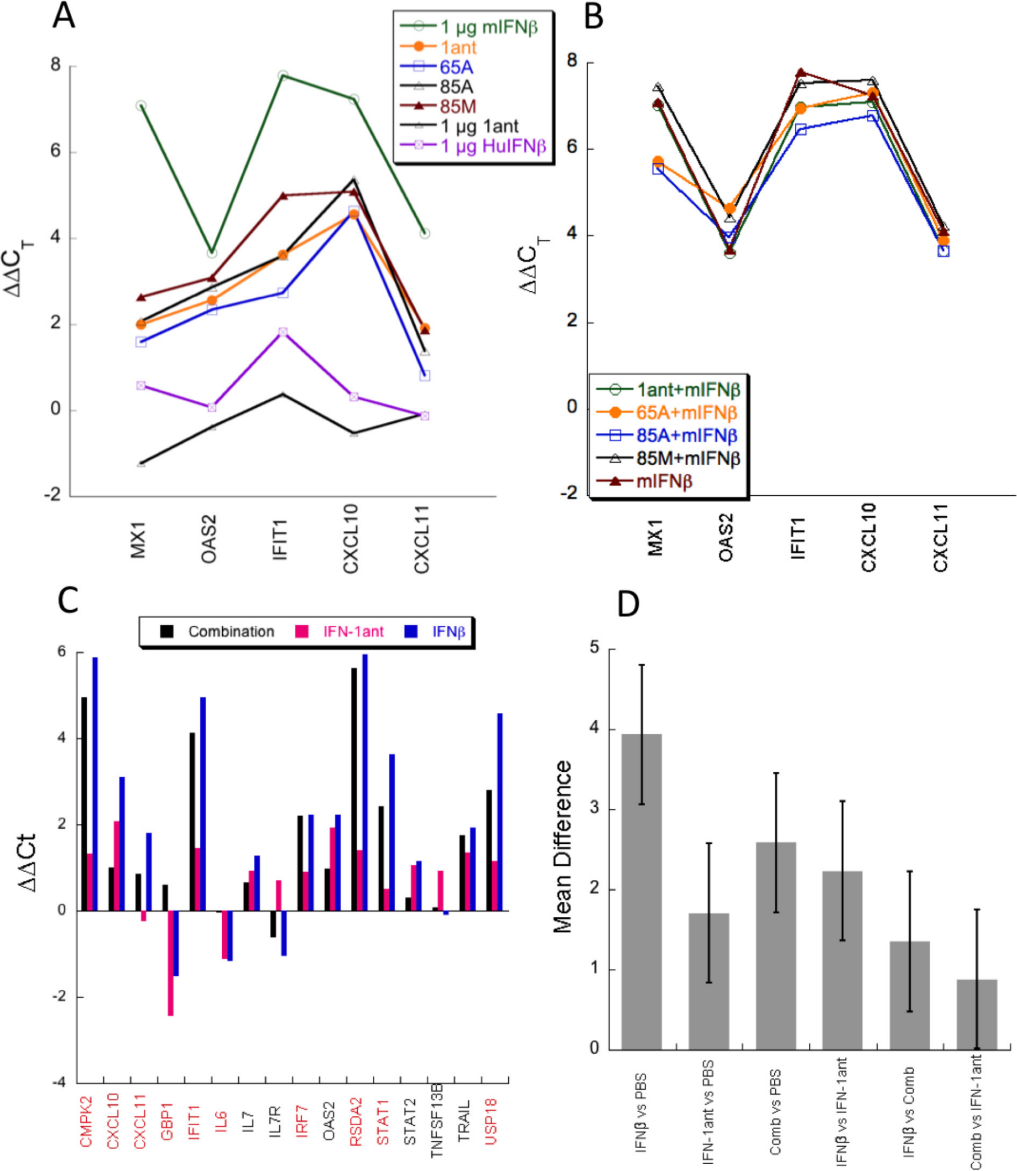
Antagonist activity in mice. Antagonist activity in mice was determined by their ability to induce or inhibit IFN-induced gene production 3 hours after injection. (A) WT C57bl/6 mice were injected with 1 μg of human (Hu) or mouse (m) IFNβ or 50 μg of each of the antagonists (1ant stands for IFN-1ant and the other antagonists are on top of the 1ant mutations). qPCR of the liver cDNA was performed to evaluate the gene induction levels of three robust (MX1, OAS2 and IFIT1) and two tunable (CXCL10 and CXCL11) genes. (B) WT C57bl/6 mice were injected I.P. with mIFNβ alone or in combination together with 50 μg of each of the antagonist. (C) HyBNAR mice were injected with 50 μg IFN-1ant, 1 μg HuIFNβ or a combination of the two. (D) Statistical analysis of the results in panel B (for genes that are differentially regulated by IFNβ versus IFN-1ant—marked in red) using Anova and Tukey post hoc test. Bars show mean difference between the treatments and error bars show 0.05 confidence intervals between the different treatments. The confidence interval for IFNβ vs combination is 0.005.

Type I interferons are species specific due to their relative low degree of sequence conservation [22]. Indeed, 1 μg of HuIFNβ is hardly elevating the level of gene induction in C57BL mice, contrary to the high level of gene induction by 1 μg of mIFNβ (Fig 8A). Even 50 μg of the antagonists injected per mouse elevate gene transcription ~10-fold less than mIFNβ (Fig 8A). Therefore, it is not surprising that neither one of the antagonists (at 50 μg/mouse) is reducing the gene induction driven by 1 μg of mIFNβ (Fig 8B). To overcome the species specificity problem we have used a transgenic mouse harboring the extracellular domains of the human interferon receptors fussed to the mouse transmembrane and intracellular domains (called HyBNAR [22]). As expected, in HyBNAR mice 1 μg of HuIFNβ elevates gene induction to almost the levels observed with 1 μg of mIFNβ (Fig 8A–8C). Conversely, 50 μg of IFN-1ant is inducing gene induction to a much lower level, in line with the cell assays shown in Fig 5. Injecting a mixture of 1 μg of HuIFNβ + 50 μg IFN-1ant to HyBNAR mice is inducing gene induction significantly less than HuIFNβ alone (Fig 8D). Overall, these results show that neither one of the antagonists is effective in wild-type mice due to species specificity, albeit they do antagonize IFN action when the human receptors are intact. Moreover, extrapolating from the cell culture results, one can assume that after longer times of activation (24 hours, instead of 3 hours in the mouse experiment) the level of inhibition of gene induction by the antagonists will be much more significant.

## Discussion

Type I interferons are best known for their role in innate immunity, but they are also involved in immunomodulation, antiproliferation, cancer surveillance, regulation of the adaptive immune response and more. Thus, interferons are general modulators as evident from the large number of genes that are regulated by IFNs (~2000) [28]. IFNs are used as therapeutic agents for a range of disease, including hepatitis C (through its antiviral functions), some classes of cancer (through its antiproliferative and immunomodulatory functions) and multiple sclerosis (which is IFNβ specific and speculated to be related to its immunomodulatory function). Conversely, excess interferon is also associated with disease, for example in lupus, tuberculosis, AIDS and cognitive decline [18–21]. In the past we have described interferon agonists, such as the YNS and YNS-α8tail, which substantially increase IFNAR1 binding (through the YNS mutations) and IFNAR2 binding (through the α8tail). We have also shown the activity profile of an interferon antagonist (IFN-1ant), which binding to IFNAR1 is reduced to below detection level by the R120E mutation. This antagonist does not activate any antiproliferative activity, its ability to induce tunable genes is very limited, but it possesses partial antiviral activity and can induce expression of robust genes [16, 21]. In this study, we further tuned the antagonist activity by adding additional mutations on top of IFN-1ant on the IFNAR1 binding surface. Using Ala scan mutagenesis we have previously determined that the single mutations N65A and Y85A reduce binding to IFNAR1 by ~3-fold. This resulted in a 6 and 30-fold reduction in the antiproliferative potency and a 1.2 and 4-fold reduction in antiviral activity of these two mutant proteins respectively. In addition, the methionine mutation at position 85 was selected as it is absence in the 500 closest homologous of IFNα2 (according to Consurf [24]). Combining these three mutations with IFN-1ant gave two variants with lower activity than IFN-1ant (N65A and Y85M) and one variant with markedly higher activity (Y85A). The later is rather surprising, as the location of Y85 on IFNα2 is remote from R120 (Fig 1A), and thus the negative cooperativity between these two residues is not easily explained.

The three new antagonists lack antiproliferative activity, even in the sensitive OVCAR3 cells. Conversely, the antiviral activity of IFN-1ant_Y85A_ is markedly higher, while that of IFN-1ant_Y85M_ and IFN-1ant_N65A_ are markedly lower than that of IFN-1ant. The same is seen for their ability to activate STATs and gene induction. Unfortunately, the direct binding affinity of either of them could not be directly determined, as it is below the detection limit of our setup (<50 μM). To further analyze the new antagonists, we looked at their ability to induce a range of genes, using the fluidigm system. In general, the same genes are induced by all the different antagonists, however to a different degree. While the slope of fold-change of gene induction of IFN-1ant versus IFN-1ant_Y85A_ is 2.7, the slope is 0.7 and 0.6 against IFN-1ant_N65A_ and IFN-1ant_Y85M_ respectively, verifying the different degree of gene activation of these 4 antagonists. Obviously, we would like also to produce an antagonist that has no interferon activity what so ever, but this seems to be very difficult, as the interferon system is adapted to transmit an antiviral signal (robust) even when the virus succeeds to block most of the interferons from binding their receptors. As receptor activation requires the formation of the ternary complex, it seems that even very transient interactions are sufficient to generate a weak signal (as seen from the STAT phosphorylation). This weak signal is amplified to produce very significant gene induction levels. Still, one should not forget that gene induction by the antagonists is observed only at nanomole protein concentrations. At this concentration the surface IFNAR2 receptors are all occupied by the antagonist (due to its sub-nM affinity to IFNAR2), and thus, some rare encounters between IFNAR1 and IFNAR2 can occur with minimal aid from the antagonist.

In summary, here we have produced and analyzed antagonists with different potencies to activate the interferon receptor. The IFN-1ant_Y85M_ and IFN-1ant_N65A_ were the weakest activators while IFN-1ant_Y85A_ was the strongest between them. None of them has antiproliferative activity, and all four of them maintain a similar antagonistic activity but with a different gene induction profile. It is difficult at this stage to say which of them would have therapeutic advantage, but they give clear choices between an antagonist that retains a higher degree of gene induction and antiviral activity and one that maintains much lower such activities.
